# Association of maternal metal exposure and psychological status with adverse pregnancy outcomes: a nested case-control study

**DOI:** 10.3389/fpubh.2025.1646045

**Published:** 2025-10-29

**Authors:** Yunyun Du, Jiashu Zhu, Chunyan Wang, Lingling Zhang, Hao Wang, Zexin Yu, Shuqin Ma, Suzhen Guan

**Affiliations:** ^1^School of Public Health, Ningxia Medical University, Yinchuan, China; ^2^Mental Health Center, General Hospital of Ningxia Medical University, Yinchuan, China; ^3^Zhuanqiao Community Health Service Center, Shanghai, China; ^4^Key Laboratory of Environmental Factors and Chronic Disease Control, Yinchuan, China

**Keywords:** maternal metal exposure, adverse pregnancy outcomes, pregnancy-related anxiety, perinatal health, nested case-control study

## Abstract

**Background:**

Uncertainties persist regarding the relationship between serum metal concentrations, maternal mental health, and adverse pregnancy outcomes. This study investigated their impact on adverse birth outcomes to improve maternal-infant health.

**Methods:**

A nested case-control study was conducted involving 468 pregnant women. Data on demographics and mental health, focusing on pregnancy-related anxiety and familial adaptation, partnerships, growth, affection, and resolve, were collected. We measured maternal serum concentrations of nine metals and trace elements. Mediator equations were used to explore the roles of maternal metal exposure and mental health in adverse pregnancy outcomes, including preterm birth, low birth weight, and large-for-gestational-age.

**Results:**

There were statistically significant differences in serum concentrations of manganese (Mn), iron (Fe), nickel (Ni), and lead (Pb) between cases (*n* = 196) and controls (*n* = 272) (*p* < 0.05). Mn, Ni, and Pb exhibited positive linear dose-responses with adverse outcomes, while chromium (Cr) indicated a negative relationship. Pregnancy-related anxiety was a risk factor (Odds ratio = 1.041, 95% confidence interval: 1.002–1.083). Strontium (Sr) had a linear relationship with pregnancy-related anxiety, whereas the other variables exhibited nonlinear associations. Maternal psychological status during pregnancy showed no mediating effect the associations between maternal exposure to Cr, Fe, Ni, and Pb and adverse pregnancy outcomes.

**Conclusion:**

The findings suggest that elevated maternal serum levels of Mn, Ni, and Pb, coupled with diminished Cr levels and increased pregnancy-related anxiety, were associated with risks of adverse pregnancy outcomes. This study establishes critical links and provides a basis for preventive strategies aimed at improving maternal and infant health.

## Graphical abstract

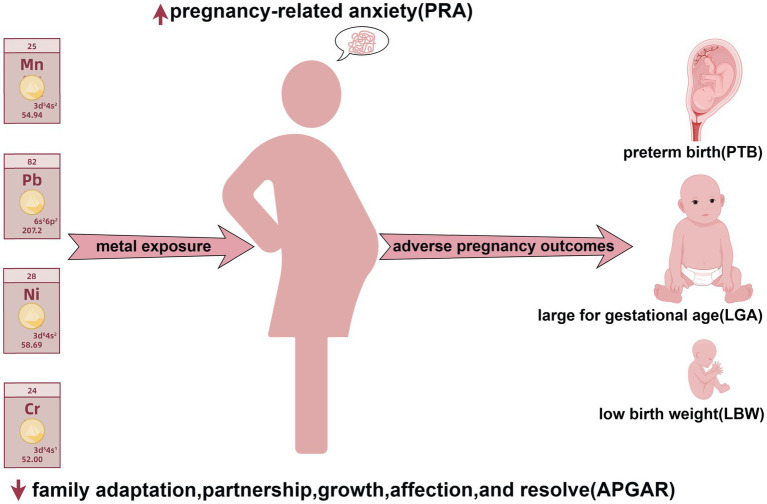

## 1 Introduction

Adverse pregnancy outcomes encompass a broad spectrum of maternal and neonatal complications, including preterm birth (PTB), low birth weight (LBW), large for gestational age (LGA), postpartum hemorrhage, and neonatal asphyxia, all of which are associated with increased risks of infant mortality, as well as long-term disease and disability (1). Among these complications, PTB and LBW are the most prevalent. In China, the overall PTB rate increased from 5.9% in 2012 to 6.4% in 2018 (2). Multiple studies have indicated that PTB is associated with an increased risk of respiratory diseases during infancy and childhood and is also closely related to altered brain development in infants and young children (3, 4). LBW is another major adverse outcome, with the World Health Organization (WHO) reporting its global annual prevalence of LBW at 15% to 20%, with a significantly higher prevalence in developing countries, which account for 95.6% of all LBW cases worldwide (5). LBW also increases the risk of cardiovascular and respiratory diseases in adulthood (6). In addition to their immediate and long-term effects on maternal and child health (7), adverse pregnancy outcomes place substantial socioeconomic burden on families, particularly regarding healthcare access and educational needs (8).

Numerous studies have explored the relationships between maternal exposure to metals and metalloids and adverse birth outcomes (9, 10). Takatani et al. (11) reported an association between maternal exposure to lead (Pb), selenium, cadmium (Cd), and manganese (Mn) during pregnancy and newborn body weight. A relatively high Mn content was positively correlated with weight loss, whereas an elevated Pb content was a risk factor for relatively low weight loss. Similarly, Hou et al. (12) reported that low serum cobalt levels were positively associated with an increased risk of LBW, whereas low serum titanium and molybdenum levels were negatively associated with this outcome. A study from Ko-CHENS in South Korea indicated that prenatal Cd exposure, even at low levels, was significantly associated with LBW (13). Nevertheless, some studies have reported no significant association between maternal metal exposure and neonatal birth weight. For example, Kim et al. (14) conducted a survey conducted in Guiyu County, China, and reported that cumulative exposure to metals [Pb, Cd, chromium [Cr], and Mn] was associated with reduced head circumference and body mass index (BMI), but not birth weight. Additionally, a study from Japan reported no associations between maternal blood mercury (Hg) levels and small-for-gestational-age status or birth weight in 15,444 pregnant women (15). These inconsistent findings highlight the need for further research to clarify the associations between maternal blood metal exposure and adverse birth outcomes in offspring.

In addition to environmental factors, the physical and mental health of pregnant women are vital determinants of pregnancy outcomes. Nevertheless, investigations into the relationship between psychological states during pregnancy and adverse pregnancy outcomes have yielded conflicting results. Dowse et al. (16) reported in a retrospective study that perinatal anxiety and depression were associated with an increased incidence of adverse birth outcomes. Several studies have suggested that adverse psychological conditions during mid-pregnancy have a more pronounced impact on birth outcomes. Mélançon et al. (17) demonstrated that women subjected to high psychological stress during mid-pregnancy have an increased risk of delivering macrosomic babies. However, other studies have identified late pregnancy as a critical period of susceptibility to adverse psychological conditions. Khalesi et al. (18) reported in a prospective cohort study that pregnancy-specific anxiety in late pregnancy was associated with preterm labor. In contrast, some studies have reported no significant associations between psychological conditions, such as anxiety and depression during pregnancy, and adverse birth outcomes. A retrospective cohort study conducted in Pennsylvania by Casey et al. (19) found no significant correlation between prenatal anxiety or depression and adverse birth outcomes. In general, although the majority of studies imply that pregnancy-related anxiety (PRA) and depression are predictors of adverse pregnancy outcomes, the precise critical period of exposure remains unclear.

Currently, data directly assessing the effects of maternal multiple metal exposures alongside mental health statuses on the risk of adverse pregnancy outcomes are scarce. This nested case-control design to investigate the mental health statuses of pregnant women attending a tertiary hospital in Ningxia. We measured the concentrations of nine metal ions in the serum of these women and collected data on the birth outcomes of their newborns. Our objective was to clarify how maternal metal exposure and mental health status during pregnancy impact adverse pregnancy outcomes, thereby providing epidemiological evidence for preventing such outcomes and improving the health of mothers and infants.

## 2 Materials and methods

### 2.1 Study population

This nested case-control study was conducted between May 2021 and May 2023, and randomly recruited 468 pregnant women who had undergone prenatal examinations at a general hospital in Ningxia were based on specified inclusion and exclusion criteria. The case group comprised women with adverse pregnancy outcomes, including LBW, PTB, and LGA infants. Eligibility criteria for the case group included: (1) aged 18–40 years; (2) gestational age exceeding 8 weeks; (3) provision of informed consent and voluntary participation; (4) permanent residency in the Ningxia Hui Autonomous Region; and (5) established personal pregnancy healthcare records at the obstetrics clinic of a tertiary hospital in Ningxia, with planned delivery at the same facility. The control group included pregnant women with healthy delivery outcomes. Participants were excluded if they met any of the following criteria: (1) prior diagnoses of mental health disorders such as depression, anxiety, bipolar disorder, or schizophrenia; or (2) inability to complete the questionnaire due to specific reasons preventing full cooperation.

### 2.2 Birth outcomes

Preterm labor was defined as delivery between 28 weeks and less than 37 weeks of gestation, categorizing the newborn as preterm. LBW was classified as a neonate weighing less than 2,500 g within the first hour after birth, with weight measured by an obstetric nurse within 5 min of delivery. LGA was defined as a gestational age above the 90th percentile. Anthropometric data—including newborn length, weight, and head circumference—were extracted from medical records.

### 2.3 Questionnaire survey

#### 2.3.1 Basic information of pregnant women during pregnancy

Basic information about the pregnant women was collected via a self-designed questionnaire. Data was collected on: (1) general demographic information (age, current residence, education level, occupation, height, pre-pregnancy and current weight, date of last menstruation, marital status, and pre-pregnancy BMI); (2) pregnancy and obstetric history (history of spontaneous abortion, mode of conception, and pregnancy attitude); (3) lifestyle factors (history of smoking and alcohol consumption, dietary habits, and sleep quality); (4) pre-pregnancy medical history (chronic diseases, mental health disorders, surgical trauma, and previous pregnancy complications); and (5) family-related factors (family living arrangements, monthly per capita income, and family history of genetic diseases).

#### 2.3.2 Psychological conditions during pregnancy

This study used the Pregnancy-related Anxiety Questionnaire (PAQ) and the Family Adaptation, Partnership, Growth, Affection, and Resolve (APGAR) index to assess family functionality and the psychological health of pregnant women in the early (≤16 weeks), middle (24–28 weeks), and late (32–36 weeks) stages of pregnancy. Women with a PAQ score of ≥24 or an APGAR score of ≤6 at any stage were considered prenatally exposed to adverse psychological conditions.

The PAQ was developed by Xiao et al. (20) from the School of Public Health, Anhui Medical University, China, and based on the experiences of pregnant women in China. The questionnaire consists of 13 items categorized into three subscales: concern about one’s own health, worry about fetal health, and worry about childbirth. The total scale and subscales demonstrated Cronbach’s alpha coefficients of 0.81, 0.64, 0.78, and 0.74, respectively, along with test-retest reliability coefficients of 0.79, 0.67, 0.75, and 0.70, respectively. The goodness-of-fit index chi-square was 14.941, indicating robust reliability and validity. According to Zhang et al. (21), scores above the 75th percentile (*P*_75_) are indicative of PRA. A score of ≥24 was used as the threshold for classifying PRA, with scores of ≥24 considered positive (indicating the presence of PRA) and scores of <24 considered negative (indicating the absence of PRA). PAQ was scored from 1 to 4, indicating “no worry,” “occasional worry,” “frequent worry,” and “always worry,” respectively, with higher scores indicating greater risks of PRA.

The APGAR, developed by Smilkstein (22) in 1978, is a self-assessment scale used to detect family function and overall satisfaction with family function. The scale consists of five items: adaptation, partnership, growth, affection, and resolve, each rated on a 3-point scale: 2 points for “Often,” 1 point for “Sometimes,” and 0 points for “Almost never.” The total score ranges from 0 to 10, with higher scores indicating better family function. Scores of 0–3 indicates severe family dysfunction, 4–6 indicate moderate dysfunction, and 7–10 reflect good family function.

#### 2.3.3 Detection of metal concentrations in blood

Assessing metal concentrations in blood is an effective method for evaluating metal exposure profiles (23). This study selected nine metals (Cr, iron [Fe], Mn, nickel [Ni], zinc [Zn], strontium (Sr), Cd, Ba, and lead [Pb]) primarily based on their relevance to maternal health during pregnancy. Fe, Zn, and Mn are essential trace elements for pregnancy, and their imbalance directly affects fetal development; Cr, Ni, Cd, and lead are environmentally hazardous metals associated with adverse pregnancy outcomes; Sr and Ba can serve as indicators of environmental exposure, reflecting the overall exposure level of the mother.

After providing informed consent, a fasting venous blood sample of 5 mL was collected from each participant by hospital professionals using vacuum tubes during routine maternity examinations, adhering to standardized aseptic procedures. The blood was subsequently centrifuged at 4 °C for 15 min at 3,000 rpm. Following separation, the serum was transferred into 2 mL cryogenic storage tubes using a 200 μL pipette and subsequently stored at −80 °C for future analysis.

Previous studies have reported methods for determining metal concentrations in serum via inductively coupled plasma mass spectrometry (ICP-MS) (24). There were some varies between the methodology employed in this study and those of other study owing to differences in our ICP-MS model and the metals measured. Briefly, an internal standard solution was prepared by diluting the internal standard (Agilent Technologies, USA) in 1% nitric acid (Sinopharm Chemical Reagent Co., China) to a final concentration of 1,000 ppb. Subsequently, 1 mL of mixed standard solution (Merck, Germany) was diluted in 1% nitric acid to achieve standard series solutions with final concentrations of 200 ppb, 100 ppb, 50 ppb, 20 ppb, 2 ppb, 1 ppb, and 0.5 ppb.

For sample preparation, serum samples were pretreated according to the protocol and placed in digestion tubes for nitrification, performed using a microwave digestion system (PerkinElmer, USA). After digestion, samples underwent drying via an acid-driving apparatus (VB24 UP, China) for 150 min before being cooled and transferred to 15 mL centrifuge tubes, with weights adjusted to 2.5 g using ultrapure water. Samples were then stored at 4 °C until analysis.

### 2.4 Statistical methods

Statistical analyses were performed using SPSS 23.0 and R 4.3.2. For serum metal levels below the limit of detection (LOD), values were imputed as LOD/^√2^. Categorical data are presented as counts (*n*) and/or percentages (%), and comparisons between groups were conducted using the chi-square test or Fisher’s exact test. Normally distributed continuous variables were expressed as mean ± standard deviation (SD); those with non-normal distributions were presented as median (M) and interquartile range (*P*_25_, *P*_75_). For normally distributed data, comparisons between groups were performed using independent-samples *t*-tests or one-way analysis of variance. Between group comparisons for non-normally distributed data were compared using the non-parametric rank-sum test (Mann–Whitney U test). The concentrations of the nine metals were skewed, and they were, therefore, logarithmically transformed and centered before analysis. A two-sided *α* = 0.05 was used as threshold for statistical significance.

Binary logistic regression was used to analyze the associations between exposure to the nine metals, adverse pregnancy outcomes, and psychological status during pregnancy. Serum metal concentrations were categorized into three levels: low (<*P*_33.3_), medium (*P*_33.3_ to <*P*_66.7_), and high (≥*P*_66.7_), with odds ratios (ORs) and 95% confidence intervals (CIs) calculated for their associations with adverse pregnancy outcomes, using the low-concentration group as the reference. Trend tests were also conducted.

Two single-metal models were developed to explore the relationships between individual metals and adverse pregnancy outcomes. Model 1 included a single metal, while Model 2 adjusted for basic demographic variables with statistically significant differences between the case and control groups, such as place of residence, pregnancy experience, and pre-pregnancy BMI.

Similarly, relationships between mental health status and adverse pregnancy outcomes were analyzed using the same methodology. Mental health status was categorized into low (<*P*_33.3_), medium (*P*_33.3_ to <P_66.7_), and high (≥*P*_66.7_) levels, with ORs and 95% CIs calculated using the low level as the reference. Trend tests were also conducted, and two models were developed to explore the relationships between maternal mental health status and adverse pregnancy outcomes.

Bayesian kernel machine regression (BKMR) models were employed to evaluate the exposure-response relationships between combined exposure to the nine metals and adverse pregnancy outcomes, adjusting for covariates such as pre-pregnancy BMI, place of residence, and pregnancy experience. Restricted cubic spline (RCS) models assessed the nonlinear dose-response relationships between combined exposure to the nine metals and psychological status during pregnancy, with concentrations log-transformed and centered for analysis. The models were adjusted for pre-pregnancy BMI, pregnancy experience, place of residence, and other psychological conditions.

In this study, the associations between combined exposure to the nine metals and psychological status during pregnancy were evaluated via a weighted quantile sum (WQS) model. Metal concentrations were log-transformed, centered, and adjusted for covariates, such as pre-pregnancy BMI, pregnancy experience, place of residence, and other psychological conditions.

## 3 Results

### 3.1 Characterization of pregnant women

The basic characteristics of the 196 cases and 272 controls included in this study are presented in Table 1. The mean ages of the participants in the two groups were 31.91 ± 4.25 and 32.11 ± 4.28 years, respectively, with no statistically significant difference (*p* > 0.05). There were significant differences in pre-pregnancy BMI, place of residence, and pregnancy experience between the two groups (*p* < 0.05). Women with low pre-pregnancy weight, residing in rural areas, and experiencing primiparity exhibited a higher probability of adverse birth outcomes. No other significant differences in baseline characteristics were observed between the two groups (*p* > 0.05).

**Table 1:** Baseline characteristics in the nested case-control study (*n* = 468).

Characteristics	*n*	Case group	Control group	*x^2^*	*p*
		*n* (%)	*n* (%)		
Age (years)					
<35	341	142 (41.6)	199 (58.4)	0.029	0.864
≥35	127	54 (42.5)	73 (57.5)		
Pre-pregnancy BMI					
<18.5	59	19 (32.2)	40 (67.8)	15.342	<0.001
18.5 ~ 23.9	303	115 (38.0)	188 (62.0)		
≥24	106	62 (58.5)	44 (41.5)		
Residence					
City	429	171 (39.9)	258 (60.1)	8.632	0.003
Countryside	39	25 (64.1)	14 (35.9)		
Educational level					
Junior high school and below	31	18 (58.1)	13 (41.9)	3.583	0.310
High school/secondary school	77	31 (40.3)	46 (59.7)		
College/undergraduate	326	133 (40.8)	193 (59.2)		
Graduate students and above	34	14 (41.2)	20 (58.8)		
Occupation					
Jobless	86	34 (39.5)	52 (60.5)	0.627	0.890
Farmers/workers	21	8 (38.1)	13 (61.9)		
Enterprises/administrative units	195	81 (41.5)	114 (58.5)		
The rest	166	73 (44.0)	93 (56.0)		
Personalities					
Introverted	44	23 (52.3)	21 (47.7)	4.097	0.129
Moderate	325	126 (38.8)	199 (61.2)		
Extroverted	99	46 (46.5)	53 (53.5)		
Pregnancy intention					
Accidental pregnancy	204	85 (41.7)	119 (58.3)	1.192	0.551
Nature	206	83 (40.3)	123 (59.7)		
Planned pregnancy	58	28 (48.3)	30 (51.7)		
Degree of early pregnancy reaction					
Mild	187	87 (46.5)	100 (53.5)	5.196	0.074
Moderate	188	79 (42.0)	109 (58.0)		
Severe	93	30 (32.3)	63 (67.7)		
Mode of conception					
Spontaneous conception	425	172 (40.5)	253 (59.5)	3.778	0.052
Artificial pregnancy assistance	43	24 (55.8)	19 (44.2)		
Household incomes per capita					
<2,000	15	5 (33.3)	10 (66.7)	1.167	0.558
2,000 ~ 5,000	231	93 (40.3)	138 (59.7)		
>5,000	222	98 (44.1)	124 (55.9)		
Pregnancy experience					
Primipara	202	98 (48.5)	104 (51.5)	6.427	0.011
Multipara	266	98 (36.8)	168 (63.2)		

BMI and body mass index are measured and defined as weight (kg)/height (m)^2^.

### 3.2 Distribution of metal concentrations in the serum of pregnant women

The serum concentrations of nine metals—Cr, Mn, Fe, Ni Zn, Sr, Cd, Ba, and Pb—of pregnant women were determined using ICP-MS. Detection limits and linear equations for each metal are presented in Supplementary Table S1, and the percentiles (*P*_5_, *P*_25_, *P*_50_, *P*_75_, and *P*_95_) are included in Table 2. Mann–Whitney U test results indicated significant differences in the concentration distributions of Mn, Fe, Ni, and Pb between the case and control groups (*p* < 0.05), with concentrations significantly lower in the control group. Fe exhibited the highest serum concentration, with a 50th percentile value of 926.19 μg/L in the case group.

**Table 2:** Concentrations of nine metals in the serum.

Metal (μg/L)	*P*_5_	*P*_25_	*P*_50_	*P*_75_	*P*_95_	*p*
Cr						0.294
Case group	7.92	9.10	9.57	10.41	12.38	
Control group	8.66	9.18	9.66	10.62	12.72	
Mn						<0.001
Case group	7.78	9.03	9.88	11.01	13.12	
Control group	6.98	7.92	9.05	10.14	12.90	
Fe						0.021
Case group	707.71	814.52	926.19	1099.56	1416.07	
Control group	652.72	787.48	882.08	1018.83	1521.08	
Ni						0.028
Case group	2.36	2.85	3.03	3.39	3.88	
Control group	2.04	2.60	2.97	3.44	3.81	
Zn						0.294
Case group	529.13	666.22	749.19	821.14	934.94	
Control group	520.46	644.64	730.11	815.56	964.68	
Sr						0.076
Case group	17.05	20.14	22.40	25.41	30.72	
Control group	16.07	18.82	21.94	25.33	31.14	
Cd						0.555
Case group	0.09	0.15	0.19	0.23	0.34	
Control group	0.07	0.14	0.18	0.22	0.43	
Ba						0.206
Case group	6.11	10.58	11.65	13.27	24.36	
Control group	5.98	9.86	11.37	13.82	18.39	
Pb						<0.001
Case group	2.07	2.53	2.77	3.05	3.81	
Control group	1.55	2.13	2.43	2.83	3.77	

### 3.3 Logistic regression analysis of maternal metal exposure and risks of adverse pregnancy outcomes

Further logistic regression analyses revealed that, before adjusting for covariates, medium (OR = 3.334, 95% CI: 2.021–5.502) and high concentrations of Mn (4.358, 3.246–8.845) significantly increased the risk of adverse pregnancy outcomes compared to low concentrations. Elevated Fe concentrations were also associated with higher risks of adverse pregnancy outcomes (1.870, 1.187–2.946). Moderate (2.843, 1.780–4.542) and high concentrations of Ni (1.682, 1.054–2.683) also increased the risk of adverse pregnancy outcomes. Compared with low concentrations, high concentrations of Sr increased the risk of adverse pregnancy outcomes (1.737, 1.100–2.743), and medium concentrations of Ba were risk factors for adverse pregnancy outcomes (2.565, 1.615–4.076); however, no trend was observed for Ba (*p* > 0.05). For Pb, medium (3.052, 1.868–4.988) and high concentrations (4.261, 2.609–6.961) increased risks of adverse pregnancy outcomes, with higher Pb concentrations associated with higher risks (*p* < 0.05).

As shown in Table 3, after adjusting for covariates such as pre-pregnancy BMI (25), residence, and pregnancy experience, medium (OR = 3.171, 95%CI: 1.899–5.295) and high concentrations of Mn (4.978, 2.978–8.320) were associated with an increased risk of adverse pregnancy outcomes compared to low concentrations. High Fe concentrations also increased the risk of adverse pregnancy outcomes (1.760, 1.100–2.818). Similarly, medium (2.947, 1.812–4.794) and high (1.748, 1.077–2.837) concentrations of Ni were associated with an increased risk of adverse pregnancy outcomes. A medium Ba concentration was a risk factor for adverse pregnancy outcomes (2.417, 1.497–3.903), but no significant trend was observed (*p* > 0.05). Compared with low concentrations, medium (3.211, 1.933–5.369) and high concentrations (4.812, 2.875–8.055) of Pb were associated with an increased risk of adverse pregnancy outcomes, with each unit increase in Pb concentrations showing a statistically significant upward trend in risk (*p* < 0.05).

**Table 3:** Association of nine metals in serum with adverse pregnancy outcomes.

Metal (μg/L)	Low	Medium	High	*p*
	–	OR (95% CI)	OR (95% CI)	
Cr	<9.330	9.330~	10.135~	
Model 1	1	0.801 (0.510, 1.257)	0.836 (0.533, 1.309)	0.433
Model 2	1	0.770 (0.483, 1.228)	0.838 (0.526, 1.333)	0.452
Mn	<8.635	8.635~	10.152~	
Model 1	1	**3.334 (2.021, 5.502)**	**5.358 (3.246, 8.845)**	<0.001
Model 2	1	**3.171 (1.899, 5.295)**	**4.978 (2.978, 8.320)**	<0.001
Fe	<834.593	834.593~	989.807~	
Model 1	1	1.281 (0.808, 2.030)	**1.870 (1.187, 2.946)**	0.007
Model 2	1	1.285 (0.800, 2.066)	**1.760 (1.100, 2.818)**	0.018
Ni	<2.828	2.828~	3.220~	
Model 1	1	**2.843 (1.780, 4.542)**	**1.682 (1.054, 2.683)**	0.037
Model 2	1	**2.947 (1.812, 4.794)**	**1.748 (1.077, 2.837)**	0.030
Zn	<687.113	687.113~	792.641~	
Model 1	1	1.574 (0.999, 2.480)	1.302 (0.826, 2.051)	0.265
Model 2	1	1.491 (0.931, 2.388)	1.339 (0.834, 2.147)	0.231
Sr	<20.338	20.338~	24.086~	
Model 1	1	1.509 (0.955, 2.385)	**1.737 (1.100, 2.743)**	0.062
Model 2	1	1.371 (0.854, 2.201)	1.571 (0.979, 2.521)	0.130
Cd	<0.159	0.159~	0.209~	
Model 1	1	0.828 (0.523, 1.309)	0.981 (0.630, 1.528)	0.945
Model 2	1	0.822 (0.511, 1.320)	0.976 (0.615, 1.549)	0.926
Ba	<10.595	10.595~	12.658~	
Model 1	1	**2.565 (1.615, 4.076)**	1.384 (0.869, 2.205)	0.194
Model 2	1	**2.417 (1.497, 3.903)**	1.300 (0.804, 2.103)	0.323
Pb	<2.371	2.371~	2.813~	
Model 1	1	**3.052 (1.868, 4.988)**	**4.261 (2.609, 6.961)**	<0.001
Model 2	1	**3.211 (1.933, 5.369)**	**4.812 (2.875, 8.055)**	<0.001

CI, confidence interval.

Bold indicates that the difference is statistically significant; Model 1 is not corrected for covariates; Model 2 is corrected for pre-pregnancy BMI, place of residence, and birth time.

### 3.4 BKMR analysis of maternal metal exposure and adverse pregnancy outcomes

**Figure 1:**
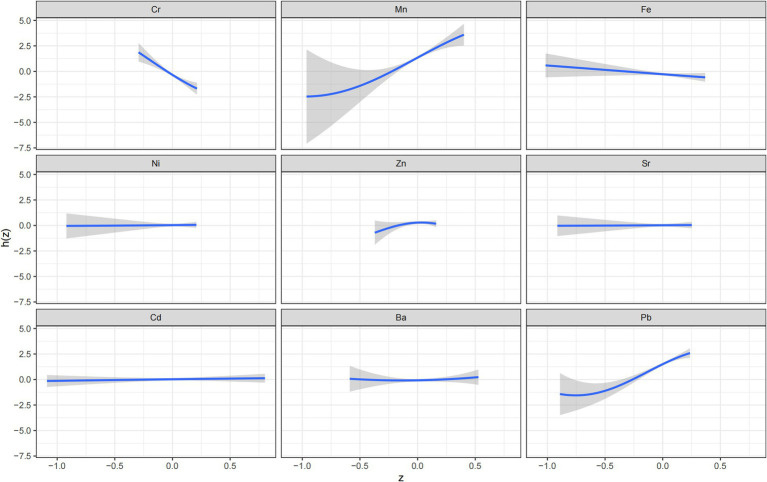
A univariate Bayesian kernel machine regression model of nine metals (Cr, Mn, Fe, Ni, Zn, Sr, Cd, Ba, Pb) and adverse pregnancy outcomes (*N* = 468).

BKMR analysis was conducted to further assess the effects of combined metal exposure on adverse pregnancy outcomes and to explore potential interactions between metals. The results indicated that, while holding the concentrations of the other eight metals constant at their median values, Cr, Fe, Ni, Sr, Cd, and Ba exhibited approximately linear relationships with adverse pregnancy outcomes. A positive exposure-response relationship was observed for Mn and Pb, indicating that the risk of adverse pregnancy outcomes increased with increasing concentrations of these metals. Conversely, a negative exposure-response relationship was identified for Cr, with increasing concentrations were associated with decreasing risk of adverse outcomes (Figure 1). Association between psychological status during pregnancy and adverse pregnancy outcomes.

Analysis revealed that 154 pregnant women (32.91%) experienced pregnancy-related anxiety. Additionally, 5 women (1.07%) had low APGAR scores indicative of severe family dysfunction, while 107 women (22.22%) exhibited moderate dysfunction. The distribution of psychological status among participants is shown in Supplementary Table S2, with PRA scores in the case group (21.70 ± 4.85) significantly higher than in the control group (20.54 ± 5.03) (*p* < 0.05). Similarly, the APGAR scores in the case group (7.81 ± 2.10) were significantly lower than those in the control group (8.45 ± 1.95) (*p* < 0.05). As illustrated in Table 4, significant differences in PRA arose among pregnant women based on their place of residence, educational level, and pregnancy intention (*p* < 0.05). Women residing in rural areas, those with lower educational attainment, or those who experienced unintended pregnancies were more likely to report PRA. Furthermore, family functioning was significantly associated with per capita income and educational level (*p* < 0.05), with higher per capita incomes and educational levels reflecting improved family functioning.

**Table 4:** Univariate analysis of the psychological status of pregnant women.

variables	n(%)	PRA (Mean ± SD)	*P*	APGAR (Mean ± SD)	*P*
Age(years)					
<35	341 (72.9)	21.26 ± 5.14	0.904	8.24 ± 2.00	0.776
≥35	127 (27.1)	20.39 ± 4.49		8.02 ± 2.13	
Pre-pregnancy BMI					
<18.5	59 (12.6)	20.92 ± 4.79	0.691	8.31 ± 1.97	0.498
18.5 ~ 23.9	303 (64.7)	20.88 ± 5.01		8.21 ± 2.03	
≥24	106 (22.6)	21.49 ± 5.02		8.03 ± 2.09	
Residence					
City	429 (91.7)	20.91 ± 4.82	0.010	8.17 ± 2.04	1.000
Countryside	39 (8.3)	22.31 ± 6.49		8.26 ± 1.94	
Educational level					
Junior high school and below	31 (6.6)	22.45 ± 6.81	0.001	7.68 ± 2.23	0.014
High school/secondary school	77 (16.5)	21.08 ± 4.89		7.57 ± 2.27	
College/Undergraduate	326 (69.7)	20.90 ± 4.79		8.34 ± 1.92	
Graduate students and above	34 (7.3)	20.76 ± 5.11		8.56 ± 2.05	
Occupation					
Jobless	86 (18.4)	21.56 ± 5.53	0.098	8.00 ± 1.88	0.686
Farmers/workers	21 (4.5)	18.57 ± 4.33		8.24 ± 2.66	
Enterprises/administrative units	195 (41.7)	21.14 ± 4.81		8.36 ± 2.01	
The rest	166 (35.5)	20.93 ± 4.92		8.06 ± 2.05	
Personalities					
Introverted	44 (9.4)	20.93 ± 4.77	0.486	7.50 ± 2.33	0.128
Moderate	325 (69.4)	21.37 ± 4.98		8.24 ± 2.02	
Extroverted	99 (21.2)	19.93 ± 4.98		8.29 ± 1.91	
Pregnancy intention					
Accidental pregnancy	204 (43.6)	23.38 ± 5.38	0.002	8.32 ± 1.90	0.560
Nature	206 (44.0)	21.07 ± 5.03		8.10 ± 2.21	
Planned pregnancy	58 (12.4)	20.31 ± 4.63		8.00 ± 1.81	
Degree of early pregnancy reaction					
Mild	187 (40.0)	20.38 ± 4.71	0.093	8.35 ± 1.85	0.397
Moderate	188 (40.2)	20.91 ± 5.06		7.99 ± 2.16	
Severe	93 (19.9)	22.57 ± 5.11		8.22 ± 2.11	
Mode of conception					
Spontaneous conception	425 (90.8)	21.11 ± 5.03	0.908	8.18 ± 2.05	0.812
Artificial pregnancy assistance	43 (9.2)	20.16 ± 4.49		8.19 ± 1.87	
Household incomes per capita					
<2000	15 (3.2)	24.33 ± 7.56	0.101	7.20 ± 2.15	0.046
2000 ~ 5000	231 (49.4)	21.44 ± 4.99		8.09 ± 2.00	
>5000	222 (47.4)	20.37 ± 4.65		8.35 ± 2.04	
Pregnancy experience					
Primipara	202 (43.2)	21.17 ± 5.01	0.783	8.21 ± 2.07	0.221
Multipara	266 (56.8)	20.92 ± 4.90		8.17 ± 2.01	

### 3.5 Association between psychological status during pregnancy and adverse pregnancy outcomes

Without adjustments for covariates, moderate and high PRA scores were associated with an increased risk of adverse pregnancy outcomes compared to low PRA scores (OR = 1.610, 95% CI: 1.001–2.589 and 1.698, 1.109–2.599, respectively) (Table 5), with a significant positive trend (*p* < 0.05). Furthermore, as a continuous variable, PRA was associated with greater risks of adverse pregnancy outcomes (1.047, 1.009–1.087; *p* < 0.05). However, no significant associations were found between the categorical or continuous APGAR scores and adverse pregnancy outcomes.

**Table 5:** Correlation analysis between psychological status and adverse outcomes during pregnancy.

Psychological condition	Model 1	Model 2
	OR (95%)	OR (95%)
PRA		
Continuous variable	**1.047 (1.009 ~ 1.087)**	**1.041 (1.002 ~ 1.083)**
Low (13~19)	1	1
Medium (20~23)	**1.610 (1.001 ~ 2.589)**	1.545 (0.944 ~ 2.530)
High (24~42)	**1.698 (1.109 ~ 2.599)**	1.550 (0.997 ~ 2.409)
*p*	0.012	0.044
APGAR		
Model	0.901 (0.785 ~ 1.033)	0.892 (0.772 ~ 1.032)
Low (0 ~ 6)	1	1
Medium (7~9)	1.024 (0.486 ~ 2.159)	1.166 (0.528 ~ 2.572)
Medium (10)	0.622 (0.294 ~ 1.315)	0.623 (0.280 ~ 1.386)
*p*	0.157	0.017

Bold indicates that differences are statistically significant; Model 1 is uncorrected for covariates; Model 2 is corrected for pre-pregnancy BMI, number of births, and residence.

After adjusting for covariates such as pre-pregnancy BMI, number of deliveries, and place of residence, PRA as a categorical variable was not significantly associated with adverse pregnancy outcomes, although PRA as a continuous variable remained a risk factor (OR = 1.041, 95% CI: 1.002–1.083), with each unit increase in PRA score associated with higher risks (*p* < 0.05). Neither categorical nor continuous APGAR scores were significantly associated with adverse pregnancy outcomes. There was a statistically significant negative trend with APGAR scores and risks of adverse pregnancy outcomes (*p* < 0.05).

### 3.6 Nonlinear dose-response relationships between metal concentrations and psychological conditions

**Figure 2:**
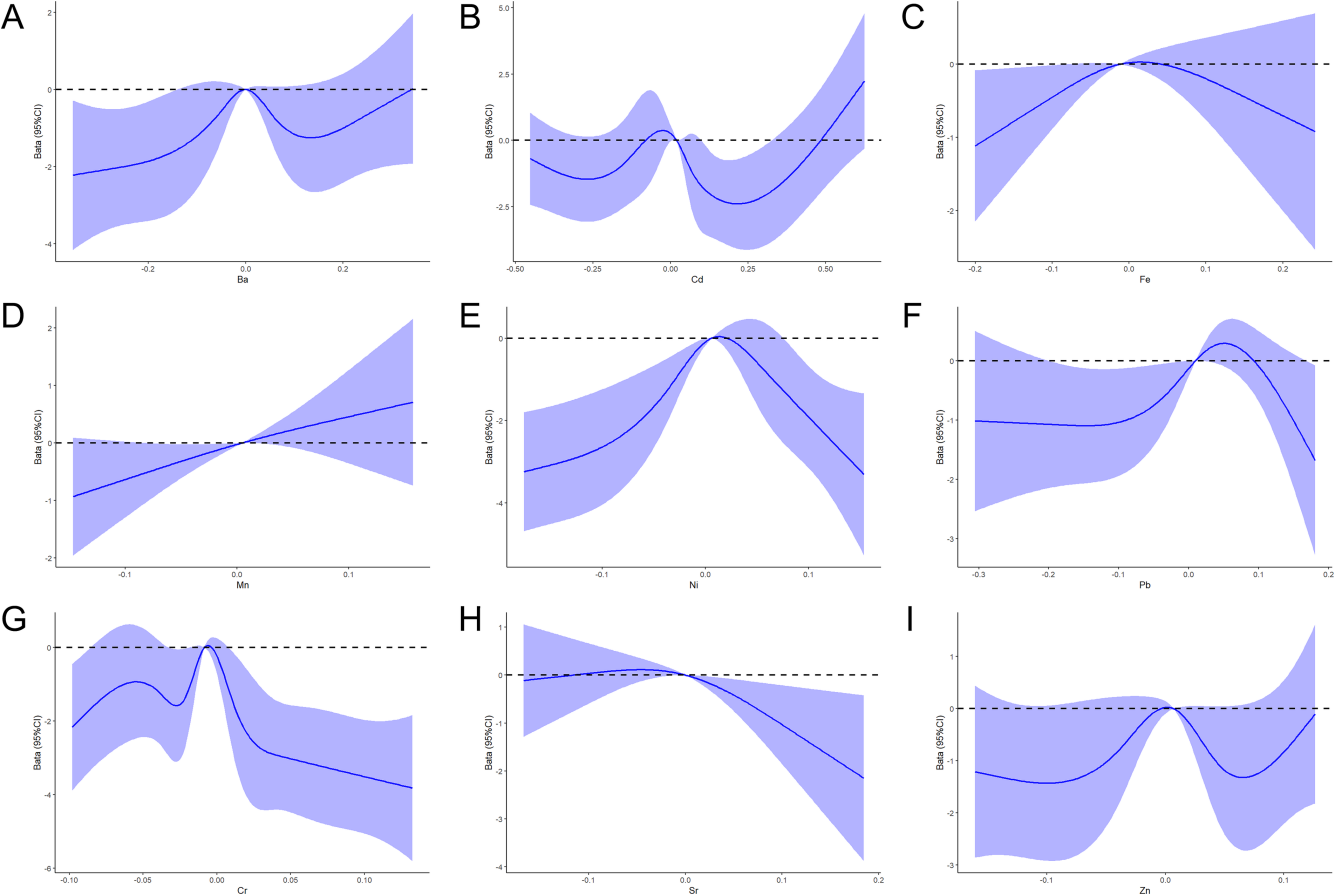
RCS function model of metal exposure and the PRA during pregnancy. **(A–I)** Nonlinear dose-response relationships between the concentrations of Ba, Cd, Fe, Mn, Ni, Pb, Cr, Sr, and Zn and the PRA.

Nonlinear dose-response relationships between concentrations of the nine metals and PRA in pregnant women were evaluated using an RCS model. Metal concentrations were log-transformed and centered, with adjustments for pre-pregnancy BMI, number of births, place of residence, and APGAR scores. As depicted in Figure 2, Cr (*P*_overall_ < 0.001, *P*_nonlinear_ < 0.001), Fe (*P*_overall_ = 0.083, *P*_nonlinear_ = 0.042), Ni (*P*_overall_ < 0.001, *P*_nonlinearity_ < 0.001), Cd (*P*_overall_ < 0.001, *P*_nonlinear_ < 0.001), and Pb (*P*_overall_ = 0.083, *P*_nonlinear_ = 0.037) exhibited significant nonlinear dose-response relationships with PRA. Sr showed a dose-response relationship with PRA (*P*_overall_ = 0.046, *P*_nonlinear_ = 0.064).

**Figure 3:**
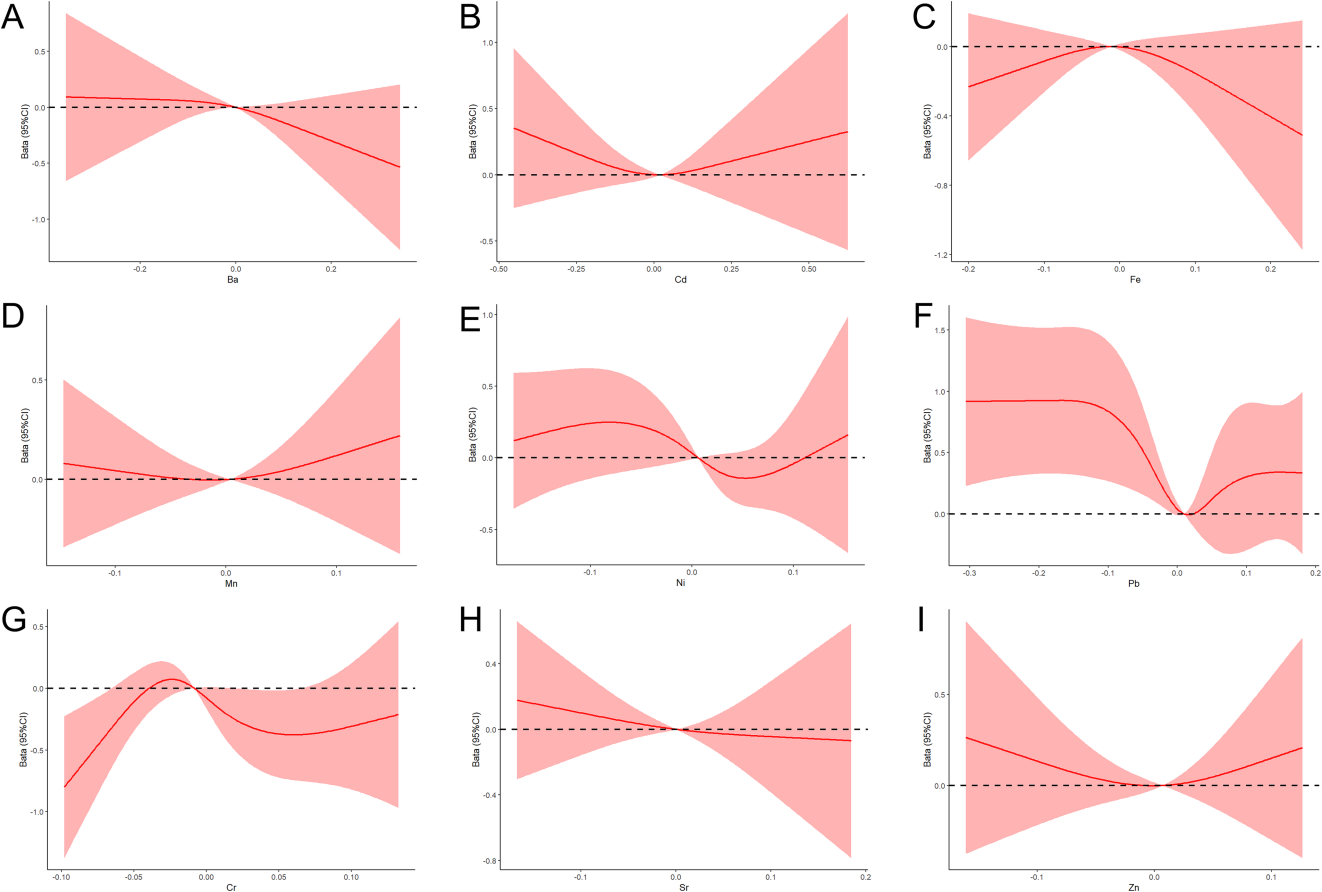
RCS function model of metal exposure and APGAR during pregnancy. **(A–I)** Nonlinear dose-response relationships between the concentrations of Ba, Cd, Fe, Mn, Ni, Pb, Cr, Sr, and Zn and APGAR.

The nonlinear dose-response relationships between the concentrations of the nine metals and the APGAR scores were also evaluated using the RCS model, with adjustments for pre-pregnancy BMI, number of births, place of residence, and PRA scores. The results indicated a nonlinear dose-response relationship between Cr and APGAR, with APGAR scores gradually increasing as Cr concentrations approached lower levels (Figure 3).

### 3.7 Mediating effect of psychological status during pregnancy on the association between maternal metal exposure and adverse pregnancy outcomes

Based on correlations between maternal metal concentrations, psychological conditions, and adverse pregnancy outcomes, as well as regression analyses between metals and adverse pregnancy outcomes, Cr, Ni, Fe, and Pb alongside PRA scores were selected for further investigation. The mediating effects of Cr and APGAR in these associations were also examined. Supplementary Table S3 illustrates that no mediating effect of psychological conditions during pregnancy was identified concerning maternal exposure to these four metals and the occurrence of adverse pregnancy outcomes.

## 4 Discussion

This study found significant differences in the overall concentration distributions of Mn, Fe, Ni, and Pb between the case and control groups. A linear dose-response relationship was observed between Mn, Ni, and Pb concentrations and adverse pregnancy outcomes. Conversely, Cr exhibited a negative relationship with adverse pregnancy outcomes, with higher Cr concentrations associated with lower risks of adverse outcomes. Additionally, no mediating effect of psychological status during pregnancy was detected in the relationship between maternal exposure to Cr, Ni, Fe, or Pb and adverse pregnancy outcomes.

Our findings indicate that the risk of adverse pregnancy outcomes increases with increasing concentrations of Mn, Ni, and Pb, which is consistent with previous studies that reported an association between exposure to metal mixtures and a greater risk of PTB (26). This association may be linked to metal-induced placental oxidative stress, epigenetic modifications, inflammation, and endocrine disruption (27). Although certain forms of Ni are known to be toxic, particularly with long-term high-dose exposure, Ni is also considered a micronutrient and is often added to foods and consumed as a dietary supplement (28). A similar trend was observed in a 2016 cross-sectional study conducted in La Palma, which reported a negative correlation between Ni levels and birth weight, with a greater risk of LBW with increasing concentration of Ni (29). However, other cross-sectional studies have shown that maternal exposure to water-soluble Ni has no significant adverse effect on pregnancy outcomes (30). This finding suggests that the exposure form and dose of Ni may be critical factors influencing its biological effects, which warrant further clarification in subsequent studies.

Mn is an essential trace element that acts as a cofactor in various biological processes, including bone formation and carbohydrate, amino acid, and lipid metabolism. Numerous studies have explored the associations between Mn levels and adverse pregnancy outcomes (31). For example, a cohort study conducted in Puerto Rico reported that high levels of Mn exposure may negatively impact birth outcomes (32), which is consistent with the findings of our study. Similarly, a case-control study in sub-Saharan Africa suggested that elevated Mn and Zn levels in the umbilical cord blood of pregnant women are associated with an increased risk of birth defects (33). Additionally, another study indicated that both high and low concentrations of Mn in maternal and cord blood may adversely affect birth size (34). This implies that there may exist a “safe window” for Mn exposure levels, and the relationship between the dynamic changes of Mn during pregnancy and fetal development merits in-depth investigation.

Early exposure to Pb, beginning prenatally and continuing throughout childhood, contributes to unhealthy and unsafe environments, particularly in industrialized countries and urban communities, where Pb pollution is a significant concern (35). Pb can cross the placenta and blood-brain barrier and accumulate in fetal tissue, causing prenatal Pb exposure associated with increased risks of PTB, LBW, and small-for-gestational-age (36). Consistent with our findings, a cohort study in Anhui demonstrated that maternal serum Pb levels during pregnancy were positively correlated with an increased risk of PTB in the Chinese population (37). Further research has demonstrated that this association may be linked to short-term (<1 week) exposure to airborne Pb during pregnancy, where even brief exposure can adversely affect birth outcomes. Reducing airborne lead exposure during pregnancy, even for a short duration, may improve birth outcomes (38). These findings provide important evidence for formulating strategies to prevent and control Pb exposure during pregnancy.

Our study revealed a 32.91% prevalence of pregnancy-related anxiety among pregnant women, comparable to rates reported in other regions. PRA was more prevalent among pregnant women residing in rural areas, those with lower literacy levels, and those with unplanned pregnancies. A study conducted in southern Minas Gerais, and Brazil, reported a 26.8% prevalence of PRA among pregnant women, with the condition being more common in late pregnancy (42.9%) (39). Similarly, a study from Bangalore, South India, reported that 55.7% of pregnant women experienced pregnancy-related anxiety disorders, with lower socioeconomic status, limited social support, and depression emerging as significant determinants of anxiety (40). Additionally, our results indicate that higher PRA score are associated with higher risks of adverse pregnancy outcomes, consistent with the findings of a cross-sectional study in Spain involving 174 pregnant women in the third trimester (41). This suggests that pregnancy-related anxiety may be a modifiable risk factor for adverse pregnancy outcomes, and targeted psychological interventions may help mitigate these risks.

Although the present study did not find a direct association between the categorical form of the APGAR score and adverse pregnancy outcomes, analysis adjusting for covariates revealed a statistically significant decrease in the risk of adverse outcomes with each unit increase in the APGAR score. This finding implies that enhanced family support or assistance during pregnancy could improve family functioning and, consequently, decrease the risk of adverse pregnancy outcomes. A study among Pacific Islander women in the United States found that 86.6% of pregnant women received assistance with daily chores, 88.1% received help during illness, and 85.1% received support for personal problems. This finding highlights the importance of strong social support in enhancing family functioning, which may positively influence perinatal outcomes (42). However, further epidemiological and experimental studies are needed to substantiate our findings concerning APGAR scores.

In this study, we found that Cr, Ni, and Cd had a nonlinear dose-response relationship with PRA, whereas Sr exhibited a linear dose-response relationship with the PRA. Additionally, Cr demonstrated a nonlinear dose-response relationship with the APGAR score. One cohort study revealed that metal exposure induces anxiety-like behaviors, with Cr and cesium having the most pronounced mixed effects (43). A series of experiments in mice and rats demonstrated that metals induce anxiety-like behaviors (44, 45), which are associated with neurochemical changes in the brain related to the pathophysiology of anxiety, such as disturbances in monoamine neurotransmitter signaling (e.g., dopamine and serotonin) (46). Another study reported that elevated Pb levels, combined with increased levels of depression, contributed to early pregnancy, decreased birth weights, and asymmetrical fetal growth (47). Collectively, these findings suggest that metal exposure may indirectly affect pregnancy outcomes by influencing psychological status during pregnancy. However, the specific dose-response patterns and regulatory mechanisms underlying this relationship remain to be elucidated through more extensive epidemiological investigations and experimental studies.

In the present study, no mediating effect of prenatal psychological status (PRA, APGAR) was identified in the association between Cr, Ni, Fe, and Pb exposure and adverse pregnancy outcomes. This result should be interpreted within the context of data characteristics and study design limitations. First, statistical power was limited; despite the 41.9% incidence of adverse pregnancy outcomes meeting nested case-control design requirements, weak correlations—such as that between Cr and PRA (*r* = 0.24) and PRA and adverse outcomes (OR = 1.041)—may have diluted observed effects. Additionally, the pathway “metal exposure-psychological status-adverse outcomes” requires significant effects across all links, but low effect sizes in certain pathways (e.g., Fe-PRA nonlinearity only significant at high levels) further constrained detection. This may relate to low serum metal levels; for instance, the 50th percentile of Pb in cases was 2.77 μg/L, far below American College of Obstetricians and Gynecologists (ACOG)’s 50 μg/L threshold for low exposure, potentially attenuating effect transmission.

Second, APGAR scores showed low variability. As an indicator of family function, 77.5% of participants scored ≥7 (indicating good function), with only 1.07% showing severe dysfunction (0–3), and a standard deviation of 2.03, indicating a “ceiling effect.” This may have attenuated associations with metal exposure (e.g., Cr-APGAR nonlinearity only significant at low concentrations) and adverse outcomes, obscuring the “metal-APGAR-outcome” pathway. Third, there was a mismatch in measurement timing and exposure windows. Metal exposure was assessed via a single serum test, while psychological status was measured across trimesters, but was defined as “abnormality at any time,” lacking a dynamic capture of psychological fluctuations. Previous research notes maternal metal effects depend on exposure timing and placental transport (48); for example, Mn/Pb peaks in late pregnancy and late-trimester PRA fluctuations (linked to PTB) may form critical mediating windows, but single-time-point measurements failed to align with these temporal factors. Finally, the scope of metals included in the mediation models was limited. The PROTECT birth cohort study demonstrated that “adverse” psychosocial states exacerbate the adverse associations between Mn and PTB, as well as between copper and small-for-gestational-age outcomes (49). However, such associations involving Mn were not observed in our study, and copper was not included in the analysis, potentially leading to the omission of relevant mediating pathways. Future work should aim to expand sample sizes, encompass high-risk groups, and include additional metals (e.g., Hg, As) to increase effect sizes. Moreover, implementing trimester-specific dynamic monitoring could facilitate the capture of temporal links, while refining APGAR subgroup analyses may reduce variability and enhance verification of psychological status as a mediator.

This study has three limitations. First, maternal serum metals were measured at a single time point, which may not fully capture exposure dynamics throughout pregnancy. Although serum levels of certain metals reflect long-term exposure in general contexts, their validity for capturing such exposure during pregnancy, given the physiological changes occurring during this period, remains uncertain. Future studies should incorporate multiple time-point sampling to better characterize exposure-outcome associations. Second, Hg, and arsenic (As) were not included due to detection constraints: serum is unreliable for Hg (short half-life, erythrocyte accumulation), and As requires LC-ICP-MS to distinguish toxicologically distinct inorganic/organic forms—both beyond the capabilities of the current study. We acknowledge the significance of these metals and aim to address this in future work via optimized sample types (e.g., whole blood, hair) and methodologies. Third, the analyses did not stratify birth outcomes by infant sex, thereby limiting exploration of fetal sex as an effect modifier in the “metal exposure-psychological status-adverse outcomes” pathway. Physiological differences between sexes may yield varied responses to maternal metal exposure, as shown in studies linking maternal red blood cell metals (As, Mn, Pb, Zn) to birth outcomes in a sex-dependent manner (50). This should be addressed in future prospective studies with dynamic stratification.

## 5 Conclusion

Using various statistical methods, this study comprehensively analyzed the associations between maternal exposure to nine individual or combined metal elements during pregnancy and adverse pregnancy outcomes. Additionally, we investigated the potential mediating role of psychological status during pregnancy in the relationship between maternal metal exposure and adverse pregnancy outcomes. These findings may provide novel epidemiological evidence for examining the intersections of metal exposure, psychological status during pregnancy, and adverse pregnancy outcomes.

## Data Availability

The raw data supporting the conclusions of this article will be made available by the authors, without undue reservation.

## References

[1] Chen R Tedroff K Villamor E Lu D Cnattingius S. Risk of intellectual disability in children born appropriate-for-gestational-age at term or post-term: impact of birth weight for gestational age and gestational age. Eur J Epidemiol. (2020) 35:273–82. doi: 10.1007/s10654-019-00590-7, PMID: 31788734 PMC7154017

[2] Deng K Liang J Mu Y Liu Z Wang Y Li M. Preterm births in China between 2012 and 2018: an observational study of more than 9 million women. Lancet Glob Health. (2021) 9:e1226–41. doi: 10.1016/S2214-109X(21)00298-9, PMID: 34416213 PMC8386289

[3] Lu MS He JR Chen Q Lu J Wei X Zhou Q. Maternal dietary patterns during pregnancy and preterm delivery: a large prospective cohort study in China. Nutr J. (2018) 17:71. doi: 10.1186/s12937-018-0377-3, PMID: 30045719 PMC6060524

[4] Boardman JP Hall J Thrippleton MJ Reynolds RM Bogaert D Davidson DJ. Impact of preterm birth on brain development and long-term outcome: protocol for a cohort study in Scotland. BMJ Open. (2020) 10:e035854. doi: 10.1136/bmjopen-2019-035854, PMID: 32139495 PMC7059503

[5] Chen Y Li G Ruan Y Zou L Wang X Zhang W. An epidemiological survey on low birth weight infants in China and analysis of outcomes of full-term low birth weight infants. BMC Pregnancy Childbirth. (2013) 13:242. doi: 10.1186/1471-2393-13-242, PMID: 24370213 PMC3877972

[6] Ueda P Cnattingius S Stephansson O Ingelsson E Ludvigsson JF Bonamy AKE. Cerebrovascular and ischemic heart disease in young adults born preterm: a population-based Swedish cohort study. Eur J Epidemiol. (2014) 29:253–60. doi: 10.1007/s10654-014-9892-5, PMID: 24687624

[7] Sacchi C Marino C Nosarti C Vieno A Visentin S Simonelli A. Association of intrauterine growth restriction and small for gestational age status with childhood cognitive outcomes: a systematic review and meta-analysis. JAMA Pediatr. (2020) 174:772–81. doi: 10.1001/jamapediatrics.2020.1097, PMID: 32453414 PMC7251506

[8] Liu D Li S Lei F Zhao Y Cheng Y Dang S. Associations between maternal calcium intake from diet and supplements during pregnancy and the risk of preterm birth in a Chinese population. Eur J Clin Nutr. (2021) 75:141–50. doi: 10.1038/s41430-020-00701-8, PMID: 32814854

[9] Zhang W Liu W Bao S Liu H Zhang Y Zhang B. Association of adverse birth outcomes with prenatal uranium exposure: a population-based cohort study. Environ Int. (2020) 135:105391. doi: 10.1016/j.envint.2019.105391, PMID: 31874351

[10] Inadera H Takamori A Matsumura K Tsuchida A Cui ZG Hamazaki K. Association of blood cadmium levels in pregnant women with infant birth size and small for gestational age infants: the Japan environment and children’s study. Environ Res. (2020) 191:110007. doi: 10.1016/j.envres.2020.110007, PMID: 32768474

[11] Takatani T Eguchi A Yamamoto M Sakurai K Takatani R Taniguchi Y. Individual and mixed metal maternal blood concentrations in relation to birth size: an analysis of the Japan environment and children’s study (JECS). Environ Int. (2022) 165:107318. doi: 10.1016/j.envint.2022.107318, PMID: 35679738

[12] Hou Q Huang L Ge X Yang A Luo X Huang S. Associations between multiple serum metal exposures and low birth weight infants in Chinese pregnant women: a nested case-control study. Chemosphere. (2019) 231:225–32. doi: 10.1016/j.chemosphere.2019.05.103, PMID: 31129403

[13] Zinia SS Yang KH Lee EJ Lim MN Kim J Kim WJ. Effects of heavy metal exposure during pregnancy on birth outcomes. Sci Rep. (2023) 13:18990. doi: 10.1038/s41598-023-46271-0, PMID: 37923810 PMC10624662

[14] Kim SS Xu X Zhang Y Zheng X Liu R Dietrich KN. Birth outcomes associated with maternal exposure to metals from informal electronic waste recycling in Guiyu, China. Environ Int. (2020) 137:105580. doi: 10.1016/j.envint.2020.105580, PMID: 32078870 PMC7257595

[15] Kobayashi S Kishi R Saijo Y Ito Y Oba K Araki A. Association of blood mercury levels during pregnancy with infant birth size by blood selenium levels in the Japan environment and children’s study: a prospective birth cohort. Environ Int. (2019) 125:418–29. doi: 10.1016/j.envint.2019.01.051, PMID: 30743147

[16] Dowse E Chan S Ebert L Wynne O Thomas S Jones D. Impact of perinatal depression and anxiety on birth outcomes: a retrospective data analysis. Matern Child Health J. (2020) 24:718–26. doi: 10.1007/s10995-020-02906-6, PMID: 32303935

[17] Mélançon J Bernard N Forest JC Tessier R Tarabulsy GM Bouvier D. Impact of maternal prenatal psychological stress on birth weight. Health Psychol. (2020) 39:1100–8. doi: 10.1037/hea0001017 33252933

[18] Khalesi ZB Bokaie M. The association between pregnancy-specific anxiety and preterm birth: a cohort study. Afr Health Sci. (2018) 18:569–75. doi: 10.4314/ahs.v18i3.14, PMID: 30602989 PMC6306999

[19] Casey JA Goin DE Rudolph KE Schwartz BS Mercer D Elser H. Unconventional natural gas development and adverse birth outcomes in Pennsylvania: the potential mediating role of antenatal anxiety and depression. Environ Res. (2019) 177:108598. doi: 10.1016/j.envres.2019.108598, PMID: 31357155 PMC6726131

[20] Xiao L Tao F Zhang L Hao J Xu S Wang H. Development and reliability evaluation of the pregnancy - related anxiety scale. Chin Public Health. (2012) 28:275–7.

[21] Zhang L Hao J Tao F Wang H Zhu P Xu S. Analysis of influencing factors of pregnancy-related anxiety in early pregnancy. Chin Public Health. (2011) 27:969–71.

[22] Smilkstein G. The family APGAR: a proposal for a family function test and its use by physicians. J Fam Pract. (1978) 6:1231–9. PMID: 660126

[23] Ashrap P Watkins DJ Mukherjee B Rosario-Pabón Z Vélez-Vega CM Alshawabkeh A. Performance of urine, blood, and integrated metal biomarkers in relation to birth outcomes in a mixture setting. Environ Res. (2021) 200:111435. doi: 10.1016/j.envres.2021.111435, PMID: 34097892 PMC8403638

[24] Kocyłowski R Grzesiak M Gaj Z Lorenc W Bakinowska E Barałkiewicz D. Evaluation of essential and toxic elements in amniotic fluid and maternal serum at birth. Biol Trace Elem Res. (2019) 189:45–54. doi: 10.1007/s12011-018-1471-2, PMID: 30097982 PMC6443612

[25] Jung CR Nakayama SF Isobe T Iwai-Shimada M Kobayashi Y Nishihama Y. Exposure to heavy metals modifies optimal gestational weight gain: a large nationally representative cohort of the Japan environment and children’s study. Environ Int. (2021) 146:106276. doi: 10.1016/j.envint.2020.106276, PMID: 33264735

[26] Liu J Ruan F Cao S Li Y Xu S Xia W. Associations between prenatal multiple metal exposure and preterm birth: comparison of four statistical models. Chemosphere. (2022) 289:133015. doi: 10.1016/j.chemosphere.2021.133015, PMID: 34822868

[27] Khanam R Kumar I Oladapo-Shittu O Twose C Islam AA Biswal SS. Prenatal environmental metal exposure and preterm birth: a scoping review. Int J Environ Res Public Health. (2021) 18:573. doi: 10.3390/ijerph18020573, PMID: 33445519 PMC7827269

[28] McDermott S Salzberg DC Anderson AP Shaw T Lead J. Systematic review of chromium and nickel exposure during pregnancy and impact on child outcomes. J Toxicol Environ Health A. (2015) 78:1348–68. doi: 10.1080/15287394.2015.1090939, PMID: 26571332

[29] Cabrera-Rodríguez R Luzardo OP González-Antuña A Boada LD Almeida-González M Camacho M. Occurrence of 44 elements in human cord blood and their association with growth indicators in newborns. Environ Int. (2018) 116:43–51. doi: 10.1016/j.envint.2018.03.048, PMID: 29649776

[30] Vaktskjold A Talykova LV Chashchin VP Odland JO Nieboer E. Maternal nickel exposure and congenital musculoskeletal defects. Am J Ind Med. (2008) 51:825–33. doi: 10.1002/ajim.20609, PMID: 18655106

[31] Hao Y Yan L Pang Y Yan H Zhang L Liu J. Maternal serum level of manganese, single nucleotide polymorphisms, and risk of spontaneous preterm birth: a nested case-control study in China. Environ Pollut. (2020) 262:114187. doi: 10.1016/j.envpol.2020.114187 32443183

[32] Ashrap P Watkins DJ Mukherjee B Boss J Richards MJ Rosario Z. Maternal blood metal and metalloid concentrations in association with birth outcomes in northern Puerto Rico. Environ Int. (2020) 138:105606. doi: 10.1016/j.envint.2020.105606, PMID: 32179314 PMC7198231

[33] Van Brusselen D Kayembe-Kitenge T Mbuyi-Musanzayi S Lubala Kasole T Kabamba Ngombe L Musa Obadia P. Metal mining and birth defects: a case-control study in Lubumbashi, Democratic Republic of the Congo. Lancet Planet Health. (2020) 4:e158–67. doi: 10.1016/S2542-5196(20)30059-0, PMID: 32353296

[34] Goto Y Mandai M Nakayama T Yamazaki S Nakayama SF Isobe T. Association of prenatal maternal blood lead levels with birth outcomes in the Japan Environment and Children’s Study (JECS): a nationwide birth cohort study. Int J Epidemiol. (2021) 50:156–64. doi: 10.1093/ije/dyaa162, PMID: 33141187

[35] Kumar S Rahman MA Islam MR Hashem MA Rahman MM. Lead and other elements-based pollution in soil, crops and water near a lead-acid battery recycling factory in Bangladesh. Chemosphere. (2022) 290:133288. doi: 10.1016/j.chemosphere.2021.133288, PMID: 34921850

[36] Sezavar AH Pourhassan B Kakavandi NR Hooshangi Shayeste MR Abyadeh M. Association of maternal blood lead concentration with the risk of small for gestational age: a dose-response meta-analysis. Arch Environ Occup Health. (2022) 77:293–300. doi: 10.1080/19338244.2021.1874857, PMID: 33492189

[37] Li J Wang H Hao JH Chen YH Liu L Yu Z. Maternal serum lead level during pregnancy is positively correlated with risk of preterm birth in a Chinese population. Environ Pollut. (2017) 1987:484–9. doi: 10.1016/j.envpol.2017.05.009 28494400

[38] Bui LTM Shadbegian R Marquez A Klemick H Guignet D. Does short-term, airborne lead exposure during pregnancy affect birth outcomes? Quasi-experimental evidence from NASCAR’S deleading policy. Environ Int. (2022) 166:107354. doi: 10.1016/j.envint.2022.107354, PMID: 35749996 PMC9829110

[39] MMJ S Nogueira DA Clapis MJ EPRC L. Anxiety in pregnancy: prevalence and associated factors. Rev Esc Enferm UP. (2017) 51:e03253. doi: 10.1590/S1980-220X2016048003253 28902327

[40] Nath A Venkatesh S Balan S Metgud CS Krishna M Murthy GVS. The prevalence and determinants of pregnancy-related anxiety amongst pregnant women at less than 24 weeks of pregnancy in Bangalore, Southern India. Int J Womens Health. (2019) 11:241–8. doi: 10.2147/IJWH.S193306, PMID: 31114392 PMC6489575

[41] Lynn FA Alderdice FA Crealey GE McElnay JC. Associations between maternal characteristics and pregnancy-related stress among low-risk mothers: an observational cross-sectional study. Int J Nurs Stud. (2011) 48:620–7. doi: 10.1016/j.ijnurstu.2010.10.002 21087767

[42] Bogulski CA Willis DE Williams CA Ayers BL Andersen JA McElfish PA. Stressful life events and social support among pregnant Marshallese women. Matern Child Health J. (2022) 26:1194–202. doi: 10.1007/s10995-022-03404-7, PMID: 35551586 PMC9095441

[43] Levin-Schwartz Y Cowell W Leon Hsu HH Enlow MB Amarasiriwardena C Andra SS. Metal mixtures are associated with increased anxiety during pregnancy. Environ Res. (2022) 204:112276. doi: 10.1016/j.envres.2021.112276, PMID: 34717944 PMC8671328

[44] Batool Z Agha F Tabassum S Batool TS Siddiqui RA Haider S. Prevention of cadmium-induced neurotoxicity in rats by essential nutrients present in nuts. Acta Neurobiol Exp. (2019) 79:169–83. doi: 10.21307/ane-2019-015, PMID: 31342953

[45] Shvachiy L Geraldes V Amaro-Leal Â Rocha I. Intermittent low-level lead exposure provokes anxiety, hypertension, autonomic dysfunction and neuroinflammation. Neurotoxicology. (2018) 69:307–19. doi: 10.1016/j.neuro.2018.08.001, PMID: 30098355

[46] Tamegart L Abbaoui A El Khiat A Bouyatas MM Gamrani H. Lead (Pb) exposure induces physiological alterations in the serotoninergic and vasopressin systems causing anxiogenic-like behavior in *Meriones shawi*: assessment of BDMC as a neuroprotective compound for Pb-neurotoxicity and kidney damages. J Trace Elem Med Biol. (2021) 65:126722. doi: 10.1016/j.jtemb.2021.126722, PMID: 33524682

[47] Appleton AA Kiley KC Schell LM Holdsworth EA Akinsanya A Beecher C. Prenatal lead and depression exposures jointly influence birth outcomes and NR3C1 DNA methylation. Int J Environ Res Public Health. (2021) 18:12169. doi: 10.3390/ijerph182212169, PMID: 34831923 PMC8620070

[48] Goldberg IB Sheiner E Bal MH Bergman D Damri NT Rosenbaum R. Early pregnancy metal levels in maternal blood and pregnancy outcomes. Sci Rep. (2024) 14:27866. doi: 10.1038/s41598-024-79107-6, PMID: 39537785 PMC11561114

[49] Ashrap P Aker A Watkins DJ Mukherjee B Rosario-Pabón Z Vélez-Vega CM. Psychosocial status modifies the effect of maternal blood metal and metalloid concentrations on birth outcomes. Environ Int. (2021) 149:106418. doi: 10.1016/j.envint.2021.106418, PMID: 33548848 PMC7897320

[50] Rahman ML Oken E Hivert MF Rifas-Shiman S Lin PID Colicino E. Early pregnancy exposure to metal mixture and birth outcomes-a prospective study in project viva. Environ Int. (2021) 156:106714. doi: 10.1016/j.envint.2021.106714, PMID: 34147999 PMC8842844

