# Supplementary material for: Association of maternal metal exposure and psychological status with adverse pregnancy outcomes: a nested case-control study
Source: Front Public Health. 2025 Oct 29;13:1646045. doi: 10.3389/fpubh.2025.1646045 (PMC12605040; doi:10.3389/fpubh.2025.1646045)
Supplement: Supplementary file 1 [file Supplementary_file_1.docx]

**Table S1 Regression equations, correlation coefficients and detection limits for serum metals in pregnant women**

| Metal element | Linear equation | Correlation coefficient r | LOD (μg/L) |
| --- | --- | --- | --- |
| Cr | Y=4.1479x+10.0208 | 1.000 | 0.339 |
| Fe | Y=4.0637x+4.4647 | 0.999 | 0.210 |
| Mn | Y=2.1354x+3.6328 | 1.000 | 0.299 |
| Ni | Y=1.7806x+1.6427 | 1.000 | 0.212 |
| Zn | Y=0.1286x+1.0725 | 0.999 | 1.818 |
| Sr | Y=0.3206x+1.6379 | 0.999 | 1.977 |
| Cd | Y=0.0741x+0.0013 | 1.000 | 0.012 |
| Ba | Y=0.1155x+0.1915 | 0.999 | 1.582 |
| Pb | Y=0.3555x+0.1282 | 1.000 | 0.277 |

**Table S2 Comparison of psychological status scores during pregnancy between the case and control groups**

| Psychological condition | Totals | Control group | Case group | t | *P* |
| --- | --- | --- | --- | --- | --- |
| PRA | 21.03±4.98 | 20.54±5.03 | 21.70±4.85 | -2.514 | 0.012 |
| APGAR | 8.18±2.03 | 8.45±1.95 | 7.81±2.10 | 3.403 | 0.001 |

**Table S3 Mediating effects of psychological status during pregnancy on the associations between maternal metal exposure and adverse pregnancy outcomes**

| Metal | Intermediary factors | Indirect effect | Direct effect | Total effect | Percentage of intermediaries(%) |
| --- | --- | --- | --- | --- | --- |
|  |  | β (95%CI) | β (95%CI) | β (95%CI) |  |
| Cr | PRA | -0.110(-0.289,0.001) | -0.408(-1.229,0.416) | -0.519(-1.434,0.319) | 21.2 |
| Ni | PRA | 0.054(-0.001,0.132) | 0.454(0.052,0.962) | 0.508(0.098,1.018) | 10.6 |
| Fe | PRA | 0.022(-0.019,0.072) | 0.302(-0.039,0.646) | 0.325(-0.029,0.659) | 6.9 |
| Pb | PRA | 0.014(-0.027,0.059) | 0.010(0.002,0.019) | 0.877(0.568,1.228) | 1.6 |
| Cr | APGAR | 0.022(-0.056,0.107) | 0.486(0.123,0.951) | 0.508(0.127,0.972) | 4.4 |
